# Lymphangioma Circumscriptum–Like Cutaneous Manifestation in Metastatic Colorectal Cancer: A Case Report Highlighting the Role of Dermatology in the Detection of Systemic Disease Progression

**DOI:** 10.1155/crdm/5173964

**Published:** 2026-10-21

**Authors:** F. A. Alghaith, R. Harby, F. AlJaber, R. Etman, H. Al-Sabah, A. AlLafi

**Affiliations:** ^1^ Asad Al-Hamad Dermatology Center Al Sabah Hospital Kuwait City Kuwait; ^2^ Jaber Al Ahmed Al Jaber Al Sabah Hospital Surra Al Asimah Governorate Kuwait

**Keywords:** colorectal cancer, cutaneous metastases, lymphangioma circumscriptum, metachronous presentation

## Abstract

Cutaneous metastases may rarely present as translucent vesicular lesions resembling lymphangioma circumscriptum (LC), posing a significant diagnostic challenge. Although colorectal cancer is among the most common malignancies globally, cutaneous metastasis remains uncommon. We present a case of a 40‐year‐old man with metastatic colorectal signet‐ring cell adenocarcinoma who presented with grouped translucent vesicles on the left lateral neck. Immunohistopathologic evaluation supported metastatic cutaneous involvement of the colorectal adenocarcinoma. This case highlights the prognostic significance of recognizing atypical cutaneous metastases and underscores the role of dermatologists in identifying disease progression through timely skin biopsy and clinicopathologic correlation. In addition, a review of previously reported solid‐tumor–associated LC‐like metastases places this presentation within the broader spectrum of metastatic cutaneous disease.

## 1. Introduction

Cutaneous metastases occasionally present as exophytic, vesicular lesions that clinically mimic lymphangioma circumscriptum (LC), creating significant diagnostic challenges in clinical practice [1]. Crucially, these lesions emerge through a distinct pathophysiological pathway involving secondary lymphatic obstruction due to regional tumor progression, which is fundamental for both diagnostic interpretation and prognostic evaluation [2]. The etiologic pathways of dermal infiltration can be explained by either lymphatic or hematogenous contiguity. In spite of such routes of infiltration, skin biopsies that confirm a metastatic cutaneous etiology are estimated at approximately 0.2% of all skin biopsies [3]. In Kuwait, such atypical dermatological presentations have necessitated increased clinical vigilance, as skin involvement often serves as a harbinger of occult or systemic progression of internal malignancy [4]. According to the latest report from Kuwait Cancer Registry, roughly 2710 cases of colorectal cancer (CRC) were documented between January 2010 and December 2019, accounting for approximately 10.2% of all malignancies in the region. Regional extension was identified in approximately 44.0% of CRC cases, whereas 27.4% of cases exhibited distant metastasis [5]. Despite such statistics, cutaneous involvement remains exceedingly rare, typically manifests in advanced disease stages following systemic progression, and can indicate a poor prognosis [6, 7].

Here, we present a 40‐year‐old male with translucent, grouped vesicles on the left neck that clinically resembled LC. However, following biopsy, histopathologic evaluation suggested cutaneous involvement of metastatic adenocarcinoma cells within the dermal lymphatics, confirming a diagnosis of cutaneous metastatic disease. The aim of this case is to highlight the necessity of histopathological analysis when cutaneous lesions masquerade as benign lymphatic malformations following a malignancy background, which can sometimes serve as the only guiding indicator for disease progression and disease prognosis.

## 2. Case Presentation

A 40‐year‐old Egyptian male with skin phototype V presented to our dermatology outpatient clinic with a cluster of asymptomatic, flesh‐colored, translucent vesicles localized to the left cervical region (Figure 1a), which had gradually progressed in size over the preceding four months since their first appearance. The lesions occurred on a background of hyperpigmented‐erythematous, mildly pruritic skin changes secondary to radiation therapy, resembling a “frogs‐spawn” appearance (Figure 1b). The vesicular eruption developed within 3 months of the radiation therapy. A full cutaneous examination revealed no other sites of similar presentation and no mucosal or genital involvement. At the time of his initial dermatologic evaluation, the lesions had been present for 3–4 months, and despite the lack of clinical response to empirical antimicrobial therapy prior to our dermatologic evaluation, the lesions continued to evolve, eventually prompting a definitive punch biopsy.

**FIGURE 1:**
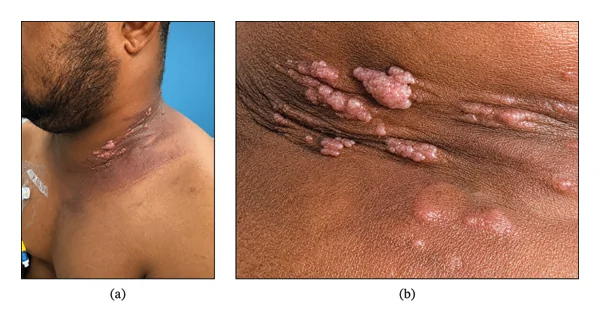
(a) Clinical picture of the flesh‐colored, translucent, fluid‐filled vesicles. (b) Close‐up of the vesicular lesions occurring on a background of erythema and mildly accentuated skin folds.

His past medical history was only significant for nonallergic asthma, newly diagnosed hypertension, and metastatic colon cancer (T4N3M1 and *KRAS* mutated) detected on a colonoscopy 2 years prior, which showed a primary fungating mass 60 cm from the anal verge in the descending colon. He successfully underwent a left hemicolectomy and three cycles of XELOX (oxaliplatin and capecitabine) during his oncologic care in Egypt. Following serial surveillance, a year and a half later, computed tomography revealed an enlarged left supraclavicular lymph node measuring 2.8 × 1.5 cm. This finding prompted further chemotherapy, bringing the total to six cycles of XELOX. His ECOG performance score was (PS = 0) (TNM stage: T4N3M1 as per previous records). He was freely ambulating and able to perform basic and instrumental activities of daily living without complications. His family history was unremarkable. He returned to Kuwait in 2026, where he was a candidate for FOLFIRI/bevacizumab chemotherapy with serial CT scans to monitor his progressive supraclavicular lymph node and underwent FNA biopsy showing nodal metastasis. His ECOG performance score had progressed to ECOG (PS: 1), with further progressive enlargement of the mediastinal lymph nodes, along with enlargement of his supraclavicular lymph node mass. He developed severe dysphagia and shortness of breath, necessitating tracheostomy in light of the threatening mediastinal and supraclavicular enlargements. Ultrasonographic evaluation of the neck demonstrated no carotid vessel involvement. A dermatology consultation was performed, and a punch biopsy specimen was obtained (3‐4 months after cutaneous onset). Following histologic confirmation, he resumed his oncologic care at the Kuwait Cancer Control Center (KCCC), where he transitioned to palliative care with palliative chemoradiation (FOLFIRI/bevacizumab + EBRT: total of 10 cycles; 10 fractions to the left supraclavicular and mediastinal lymph nodes), leading to improvement in airway compression on follow‐up imaging. Despite an initially unremarkable fiberoptic laryngoscopy, repeat examination later showed sluggish vocal cord mobility. Due to severe dysphagia, total parenteral nutrition (TPN) was commenced, and oral intake was withheld. Laboratory evaluation demonstrated mild microcytic anemia, hypoalbuminemia, and a mild isolated GGT elevation, with otherwise preserved renal, hepatic, electrolyte, and coagulation profiles. Baseline testing for HIV, TB, and other HIV defining infections was negative.

Given the systemic progression, the clinical focus shifted to palliative care using chemoradiation, and the patient pursued his care back in his country of origin.

A punch biopsy specimen from the representative neck lesion was obtained and routinely processed for H&E staining and immunohistochemical profiling. A histopathologic examination revealed dermal infiltration by malignant cells arranged in diffuse sheets (Figure 2). The tumor cells demonstrated markedly pleomorphic nuclei with prominent nucleoli and frequent mitoses and extensive foci of lymphovascular invasion coupled with lymphatic vessel dilation. Immunohistochemistry showed diffuse CK20 positivity, patchy CDX2, and focal CK7 positivity and expression (Figure 3), supporting metastatic colorectal adenocarcinoma. C2‐40 staining was performed to support the lymphovascular dilation and invasion, simulating LC‐like findings.

**FIGURE 2:**
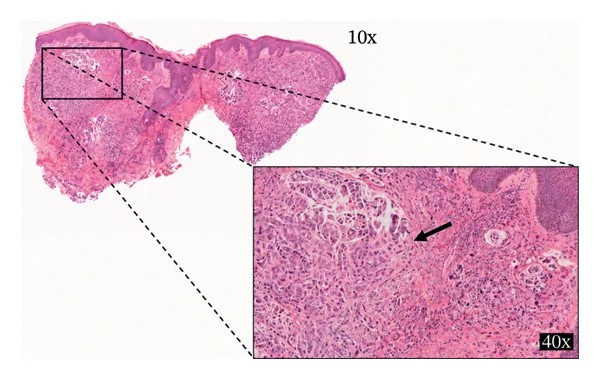
Skin biopsy revealing colorectal adenocarcinoma involving the skin with dermal infiltration by malignant cells arranged in diffuse sheets (arrow). Marked pleomorphic nuclei with prominent nucleoli, frequent mitoses, and extensive foci of lymphovascular invasion (inset, 40x).

**FIGURE 3:**
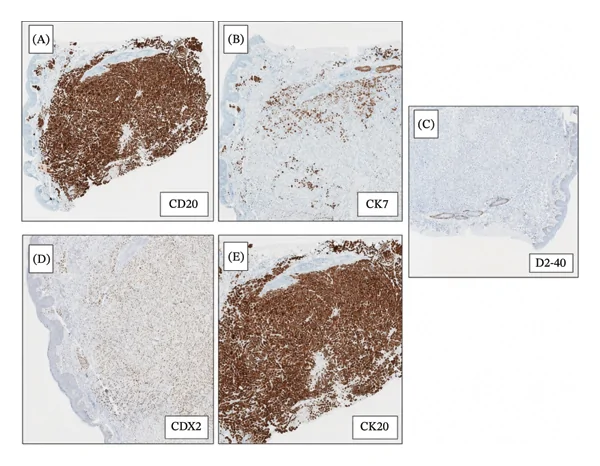
Histopathologic and immunohistochemical evaluation of a lymphangioma circumscriptum–like cutaneous metastasis from colorectal adenocarcinoma. (A) CD20 demonstrates strong, diffuse positivity. (B) CK7 shows focal positivity. (C) D2‐40 highlights the endothelial lining of dilated lymphatic channels, supporting lymphatic involvement in conjunction with the histopathologic findings. (D) CDX2 demonstrates patchy nuclear expression. (E) CK20 shows strong, diffuse positivity.

## 3. Discussion

Cutaneous metastasis originating from colorectal carcinoma is a rare phenomenon, with reported incidence rates typically ranging between 2.3% and 6.0% [8, 9]. High‐level evidence from the most extensive systematic review and meta‐analysis conducted by Tokarski et al. [10] further confirms that cutaneous metastases are uncommon and serve as surrogate markers or “sentinels” for aggressive systemic disease. While some reports indicate an incidence rate of fewer than 5% of metastatic cases [11], recent clinical reviews support the broader 2%–6% range as the standard for colorectal carcinoma [8]. Here we report a case of metachronous presentation of LC‐like lesions occurring in a 40‐year‐old patient, who was previously managed with surgical resection combined with adjuvant radio‐chemotherapy.

Atypical cutaneous presentations described in colorectal and rectal adenocarcinoma include inflammatory or erysipeloid plaques, zosteriform eruptions, herpetiform or pseudovesicular lesions, cystic/subcutaneous deposits, ulcerated or fungating lesions, and umbilical metastasis (Sister Mary Joseph nodule); genital, vulvar, penile, scalp, facial, and other unusual sites have also been reported in the literature [12–15]. Cutaneous metastases may precede, coincide with, or follow the diagnosis of the primary malignancy. In the largest colorectal‐specific clinicopathological series, 52% of CM were synchronous with CRC (defined as occurring within 12 months of diagnosis), whereas 48% were metachronous (> 12 months) [13].

Given the clinical challenge of identifying the lesions, the differential diagnoses include primary LC, eruptive lymphangioma, irritant contact dermatitis or, in this context, metastatic carcinoma masquerading as inflammatory dermatoses.

The immunohistochemical profile from the obtained skin biopsy, specifically CDX2 and CK20 positivity, is a valuable tool for confirming the colorectal origin of such metastatic adenocarcinomas [16]. The immunophenotypic profile (e.g., CK20‐positive, CDX2‐positive, and focally CK7‐positive) in conjunction with the patient’s known *KRAS*‐mutated signet cell colorectal adenocarcinoma supports the diagnosis of cutaneous metastatic colorectal adenocarcinoma with lymphatic spread, producing a clinical presentation of LC‐like or acquired lymphangectasia‐like morphology (Figure 1b). While CDX2 and CK20 positivity support colorectal origin, it should be emphasized that CK7 positivity is often seen in specimens from the lung, breast, ovary, and upper digestive tract, while tumors of the colon, prostate, and kidney are usually CK7‐negative. However, its positivity in colorectal biopsies portends a poor prognosis [17].

LC is a benign lymphatic malformation due to congenital or acquired lymph vessel ectasia, resulting in the characteristic superficial, dilated, and thin‐walled vesicles observed in the papillary component of the dermis. The pathogenesis involves primitive lymphatic sacs, which fail to connect to the central lymphatic system, leading to sequestration as deep subcutaneous cisterns according to Whimster’s hypothesis [18, 19]. This theoretical framework posits that these disconnected lymphatic cisterns maintain communication with the superficial dermal plexus through vertical channels, eventually undergoing dilatation to form the classic “frog‐spawn” appearance (Figure 1a) seen in LC [20]. In lymphangioma‐like lesions that are due to lymphatic obstruction, a comparable morphological picture can be appreciated, especially in metastatic disease [20]. Therefore, adult‐onset LC‐like lesions, especially in the context of internal malignancy, should not be assumed to be benign and warrant further urgent investigation for possible restaging.

Vesicular and pseudovesicular phenotypes are extraordinarily rare. The body of literature surrounding CRCs does not explicitly reflect a *bona fide* LC‐like phenotype. Most reported morphologies are nodular or plaque‐like, with atypical inflammatory variants also described [4]. The nearest case resembling an LC‐like morphology was described by Jing et al. [21]. However, superficial dermal lymphatic invasion has been reported previously.

A review of literature exploring the prognostic stratification of the timing of cutaneous lesions in relation to CRC patients’ remaining life trajectory found that synchronous presentation (e.g., metastatic cutaneous presentation at the time of their primary diagnosis) signified aggressive underlying tumor biology and strongly correlated with advanced‐stage systemic involvement and an overall poor clinical prognosis [22–24], whereas metachronous presentations may emerge after a disease‐free period during surveillance. Those with metachronous presentations had a significantly better median survival of 12 months. They sometimes appear as solitary recurrences, which distinguishes them from the widely disseminated disease seen in synchronous cases. In instances of such isolated cutaneous deposits, radical surgical excision may serve as a viable, life‐extending strategy, possibly conferring a survival advantage over palliative care alone and reinforcing the necessity for rigorous, ongoing dermatological monitoring in long‐term CRC survivors [22]. Regardless of the temporality, the appearance of skin metastases generally portends a dismal prognosis, often marking the terminal stage of metastatic colorectal disease [11]. A meta‐analysis performed in 2025 reviewing cutaneous metastases associated with CRCs found that these lesions typically manifest as a sign of late‐stage systemic progression, often foretelling a poor prognosis due to widespread lymphovascular involvement [10].

The role of dermatology in the management of CRC is often underestimated, yet the “*dermatologist’s eye*” can serve as a pivotal point in systemic restaging. By maintaining a high index of suspicion and utilizing skin biopsies in metastatic workup, dermatologists can detect and recognize systemic progression early [22, 25]. Given the diagnostic challenges posed by mimickers such as LC, clinicians should consider cutaneous markers as potential sentinel events for systemic disease progression [10]. Early identification and recognition of these cutaneous findings can provide quantifiable benefits when revising treatment decisions.

While the median survival time between detection of these cutaneous findings and death was reported to be 5.5 months [10], radical management of isolated cutaneous metastasis can extend a patient’s median survival by nearly 10 months beyond what is achieved with palliative chemotherapy alone and postdetection average lifespan. In some instances, this proactive approach has resulted in disease‐free intervals of 12 months or more [22, 26]. In a large series of 29 metastatic cutaneous lesions in colon cancer, facial skin lesions were only raised in two out of five cases, highlighting the rarity of such anatomical presentations in the context of disseminated systemic malignancy and the higher chances of misdiagnosis [4].

A recent case report described a patient who developed subcutaneous nodules 2 years after their primary colon cancer diagnosis. The nodular lesions appeared approximately 3 months before liver and lung metastases [27]. This “lead‐time” provided by the skin highlighted the role of dermatology not only as a diagnostic consultation but also as a proactive discipline that could trigger the need for systemic restaging. When dermatologists identify such metachronous lesions—which in the cited case served as the first sign of recurrence—they provide a vital window for clinicians to escalate care before the disease reaches a terminal, multiorgan stage [27]. As in the case of our patient, a 3‐4‐month lead‐time between his cutaneous findings and the shift to palliative care was instrumental in facilitating a re‐evaluation of systemic therapy, thereby highlighting how cutaneous manifestations can act as critical data for internal disease recurrence [28, 29]. As in other cases, our case further corroborates the larger body of evidence relating to the prognostic stratification suggested above, where the detection of metachronous lesions during the surveillance period is associated with a more favorable median survival compared to synchronous presentations [22].

By recognizing cutaneous metastases as sentinel events, the dermatologist can trigger immediate PET‐CT staging, potentially identifying isolated recurrences that are amenable to radical, life‐extending surgical excision rather than immediate transition to palliative care [22, 27]. Consequently, incorporating a thorough dermatological evaluation into routine follow‐up protocols for CRC surveillance is essential, even in the absence of obvious symptoms [8, 30]. With respect to the complex prognostication process in CRC, dermatology providers serve as a critical diagnostic link in multidisciplinary care delivery, ensuring that cutaneous findings in patients with histories of visceral malignancy are scrutinized rather than overlooked [4, 31]. Early detection remains imperative, as approximately 18.6% of patients presenting with cutaneous metastases are initially identified as having a previously silent primary malignancy.

## Funding

No funding was received for this research.

## Ethics Statement

Institutional review board approval was not required for this single case report. Informed consent for research publication of clinical information and images was obtained prior to the production and presentation of this case.

## Consent

Please see the Ethics Statement. Informed consent to use personal data for the publication of this article and any accompanying images was obtained. All patient information in the case report was appropriately de‐identified.

## Conflicts of Interest

The authors declare no conflicts of interest.

## Supporting Information

Additional supporting information can be found online in the Supporting Information section.

## Supporting information


**Supporting Information** CARE checklist.

## Data Availability

The data supporting the findings of this study are available from the corresponding author upon reasonable request.
