# Supplementary material for: Lymphangioma Circumscriptum–Like Cutaneous Manifestation in Metastatic Colorectal Cancer: A Case Report Highlighting the Role of Dermatology in the Detection of Systemic Disease Progression
Source: Case Rep Dermatol Med. 2026 Oct 21;2026:5173964. doi: 10.1155/crdm/5173964 (PMC13685503; doi:10.1155/crdm/5173964)
Supplement: Supplementary file 1 [file CRDM-2026-5173964-s001.pdf]

CARE case report guidelines

CARE Checklist of information to include when writing a case report

(cc) BY-NC-ND

Topic	Item	Checklist item description	Reported on Line
Title	1	The diagnosis or intervention of primary focus followed by the words "case report"	1-4
Key Words	2	2 to 5 key words that identify diagnoses or interventions in this case report, including "case report"	39-40
Abstract (no references)	3a	Introduction: What is unique about this case and what does it add to the scientific literature?	45-55
	3b	Main symptoms and/or important clinical findings	49-53
	3c	The main diagnoses, therapeutic interventions, and outcomes	53-56
	3d	Conclusion—What is the main "take-away" lesson(s) from this case?	56-59
Introduction	4	One or two paragraphs summarizing why this case is unique (**may include references**)	60-84
Patient Information	5a	De-identified patient specific information.	85-125
	5b	Primary concerns and symptoms of the patient.	85-93
	5c	Medical, family, and psycho-social history including relevant genetic information	95-124
	5d	Relevant past interventions with outcomes	98-124
Clinical Findings	6	Describe significant physical examination (PE) and important clinical findings.	85-93
Timeline	7	Historical and current information from this episode of care organized as a timeline	85-127
Diagnostic Assessment	8a	Diagnostic testing (such as PE, laboratory testing, imaging, surveys).	128 -136
	8b	Diagnostic challenges (such as access to testing, financial, or cultural)	91-93;147-157
	8c	Diagnosis (including other diagnoses considered)	126-134;146-154
	8d	Prognosis (such as staging in oncology) where applicable	99-118;135-145
Therapeutic Intervention	9a	Types of therapeutic intervention (such as pharmacologic, surgical, preventive, self-care)	n/a
	9b	Administration of therapeutic intervention (such as dosage, strength, duration)	n/a
	9c	Changes in therapeutic intervention (with rationale)	n/a
Follow-up and Outcomes	10a	Clinician and patient-assessed outcomes (if available)	119-125;200-223
	10b	Important follow-up diagnostic and other test results	119-124; n/a
	10c	Intervention adherence and tolerability (How was this assessed?)	n/a
	10d	Adverse and unanticipated events	n/a
Discussion	11a	A scientific discussion of the strengths AND limitations associated with this case report	not reported,discussed
	11b	Discussion of the relevant medical literature **with references**.	137-227
	11c	The scientific rationale for any conclusions (including assessment of possible causes)	228-237
	11d	The primary "take-away" lessons of this case report (without references) in a one paragraph conclusion	229-237
Patient Perspective	12	The patient should share their perspective in one to two paragraphs on the treatment(s) they received	not reported
Informed Consent	13	Did the patient give informed consent? Please provide if requested	Yes ☑ No ☐
